# Comparative cross-sectional study of ultrasonography and thyroid scintigraphy findings in adult patients with nodular goiter

**DOI:** 10.1097/MD.0000000000042019

**Published:** 2025-04-25

**Authors:** Fares Abboud, Hind Alsiddig, Sultaneh Haddad, Basil Daradkeh, Ghassan Bayat, Farah Haneyah

**Affiliations:** a Damascus University, Faculty of Medicine, Damascus, Syrian Arab Republic; b Nile University, Faculty of Medicine, Khartoum, Sudan; c Division of Pediatrics, Children’s Hospital, Damascus, Syrian Arab Republic; d Jordan University of Science and Technology, Faculty of Medicine, Irbid, Jordan; e Plymouth University Hospitals NHS Trust, Plymouth, United Kingdom; f Al-balqa Applied University, As-Salt, Jordan.

**Keywords:** blood flow indices, endocrinology, nodular goiter, scintigraphy, thyroid, ultrasonography

## Abstract

Thyroid nodular goiter is a prevalent condition requiring imaging for diagnosis and management. Ultrasonography and thyroid scintigraphy are commonly used modalities, but their comparative diagnostic roles remain unclear. To compare ultrasonography and thyroid scintigraphy findings in nodular goiter patients, evaluate correlations between thyroid uptake and blood flow indices, and assess the role of imaging in determining thyroid and nodule size. A cross-sectional observational study conducted from January to November 2021 at the radiation and isotopes center Khartoum. The study included 100 adult patients (86% female, 14% male) with nodular goiter, aged 19 to 70 years (mean: 39.26 ± 14.19 years), randomly selected. Ultrasonography was performed using a high-frequency linear transducer (6–15 MHz) to evaluate thyroid and nodule size, echogenicity, texture, and blood flow indices (resistive index, PI, PSV). Scintigraphy utilized technetium-99m to classify thyroid gland uptake (high, normal, low). Data were analyzed using SPSS; *P* < .05 was considered significant. Demographics: most participants were aged 19 to 35 (46%) and married (76%). Isoechoic nodules were most common (68%), with 60% showing heterogeneous texture. High uptake was observed in 74% of patients, normal in 10%, and low in 16%. Significant differences were found across uptake categories for resistive index (*P* = .00), PI (*P* = .00), and PSV (*P* = .005). A moderate positive correlation (*R*² = 0.4075) existed between thyroid size and nodule size. Uptake did not significantly affect thyroid or nodule size. Ultrasonography provided detailed structural and vascular data, while scintigraphy evaluated gland-wide functional activity. Ultrasonography and scintigraphy complement each other in managing nodular goiter. Combining modalities enhances diagnostic accuracy and treatment planning.

## 1. Introduction

Thyroid disorders are among the most prevalent endocrine conditions globally, affecting an estimated 20% to 70% of individuals depending on the population studied.^[1]^ A variety of symptoms from these conditions impact people across different age groups, with the most frequent ones being goiter, Grave disease, and Hashimoto disease. Nodular goiter is the presence of one or more nodules within the thyroid gland. Early and accurate diagnosis of these nodules is critical to guide management, ranging from conservative follow-up to surgical intervention. Imaging modalities play a central role in this diagnostic process, providing essential insights into nodule characteristics, thyroid gland morphology, and functional status.^[2]^

Thyroid nodules are commonly found by chance during computed tomography, magnetic resonance imaging, or positron emission tomography scans, but ultrasonography (US) is broadly considered the primary method for assessing the thyroid due to its accessibility, noninvasive nature, and ability to provide high-resolution structural details. Doppler US further enhances its utility by assessing blood flow indices, which may correlate with thyroid nodule activity and vascularity.^[3]^ In contrast, thyroid scintigraphy using technetium-99m (Tc-99m) pertechnetate offers functional insights by evaluating radiotracer uptake patterns across the gland. High uptake concentrated in a specific area often suggests hyperfunctioning (hot) nodules, while low uptake is typically associated with hypofunctioning (cold) nodules, potentially indicating malignancy.^[2]^

Despite their complementary roles, the comparative diagnostic utility of ultrasonography and thyroid scintigraphy remains an area of ongoing research. Ultrasonography’s detailed structural evaluation has raised questions about whether it could replace scintigraphy in certain scenarios, particularly in resource-limited settings.^[1,3]^

This study aims to address the gap by evaluating and comparing ultrasonographic and scintigraphic findings in patients with nodular goiter. Specifically, it seeks to assess correlations between imaging characteristics, thyroid uptake patterns, and blood flow indices, providing insights into their diagnostic accuracy and reliability. By exploring the strengths and limitations of these modalities, this study contributes to optimizing diagnostic strategies for thyroid nodules, particularly in resource-constrained environments.

### 1.1. Problems of the study

In Sudan, thyroid goiter cases have recently surged due to multiple contributing factors. Relying solely on thyroid hormone levels isn’t a dependable or precise method for diagnosing thyroid conditions. The country faces a scarcity of facilities for thyroid studies, limited public knowledge about the issue, and delayed patient visits often occurring in advanced stages of thyroid disorders. In nuclear medicine departments, radiation protection measures are inadequately implemented when administering radioactive substances, even for what’s considered a “small radiation dose.” This leads to neighboring tissues receiving the same radiation exposure as the thyroid because dosage adjustments are overlooked. Ultrasonography could take on a primary role in monitoring patients over time, potentially replacing scintigraphy.

## 2. Objectives of the study

### 2.1. General objective

The general objective is to study compressions between ultrasonography and scintigraphy findings in goiter patients.

#### 2.1.1. Specific objectives of the study

- To examine how ultrasound results stack-up against scintigraphy outcomes and figure out if ultrasound by itself is enough to diagnose thyroid nodular goiter.
- To determine the thyroid’s dimensions through the use of ultrasound.
- To evaluate the thyroid’s absorption levels and blood flow indices.
- To investigate the relationship between thyroid texture, echogenicity, and shape using ultrasound and scintigraphy, alongside the connection between thyroid uptake and blood flow measure.

#### 2.1.2. Previous studies

- Research from Hahn et al. (2004)^[1]^ indicates that high-resolution ultrasonography (US) has enabled the identification of small, symptomless thyroid nodules. These thyroid incidentalomas pose a challenge in determining the best way to handle such unexpected discoveries. The study explored their frequency, clinical and US features, and the most effective diagnostic method for identifying benign versus malignant thyroid nodules under 1.5 cm. A retrospective analysis included 1475 patients who attended Samsung Medical Center in Seoul, Korea, between January 1999 and December 2000. Thyroid incidentalomas were found in 13.4% of cases, with 28.8% of these being malignant. No notable differences appeared in nodule size, patient age, gender, thyroid function tests, or Tc99m thyroid scans between benign and malignant incidentalomas. However, US traits like solid echostructure, irregular edges, and calcification were significant indicators of malignancy (*P* < .05). Most malignant incidentalomas were early-stage. In summary, hidden thyroid cancers are relatively frequent, and while clinical and lab data showed no major distinctions between benign and malignant nodules under 1.5 cm, US characteristics can guide the best management approach.^[1]^
- A study conducted by Hsiao et al. (1994)^[2]^ explored how thyroid ultrasonography and/or aspiration cytology, alongside thyroid hormone and antibody tests, are typically used, though palpation might not effectively spot thyroid issues. The goal was to assess how common thyroid irregularities are among Chinese adults undergoing health checkups, as identified by ultrasonography. The research involved 277 individuals (ages 17–79, averaging 52 years) who visited National Taiwan University Hospital for routine exams. Out of these, 200 with goiter received thyroid ultrasonography, revealing abnormalities in 37 cases (18.5%). The study also measured thyroid volumes via ultrasonography, finding an average of 7.7 ± 3.3 mL, which showed a positive link to body mass index (*R* = 0.17, *P* < .05) and body weight (*R* = 0.28, *P* < .005). Men (n = 115) exhibited greater thyroid volumes than women (n = 48), with measurements of 8.3 ± 3.3 mL versus 6.1 ± 2.6 mL (*P* < .001).^[2]^
- Research by Mohammed (2017)^[3]^ involved a descriptive cross-sectional analysis primarily carried out at the radiation and isotopes centre of Khartoum Medical Diagnostic Center, Academy Charity Teaching Hospital, and Bashair Teaching Hospital from May to December 2016. The study included 50 patients of both genders, examined with ultrasound and Technetium-99m radionuclide scans, all performed in a supine position. Data analysis was conducted using the Statistical Package for Social Science (SPSS), revealing that benign thyroid nodules were more frequent in females (86%), particularly among married individuals (76%), with ages ranging from 22 to 70 years. These nodules were less common in younger and older patients, predominantly bilateral (62%), showed high uptake (74%), and had a mostly homogeneous radiotracer distribution (62%) with regular outlines (60%). Ultrasound findings indicated 60% heterogeneous texture, with echogenicity being 60% isoechoic, 20% hypoechoic, and 12% hyperechoic; 90% of nodules had well-defined outlines, while 10% were ill-defined. The study linked nuclear medicine results with ultrasound observations, finding no notable differences in thyroid or nodule volume across high, low, or normal uptake groups via ANOVA testing (*P* = .05), suggesting thyroid uptake doesn’t influence volume. However, blood flow indices (PI, RI, PSV) showed significant effects for RI and PSV in the same uptake groups, indicating blood flow reflects thyroid function. A connection between thyroid nodule volume and overall thyroid volume was also noted. Mohammed (2017) concluded that while scintigraphy offers functional insights, it lacks precision compared to ultrasound, especially Doppler-enhanced ultrasound, which provides a more detailed and dependable assessment of nodule features and blood flow. The study highlights the need to improve ultrasound technology for better diagnostic accuracy.^[3]^

## 3. Materials and methods

### 3.1. Materials

Ultrasound machines with liner array (7–10 MHZ), coupling gel, laser printer, weight and length devices, and gamma camera machines using technetium pertechnetate.

### 3.2. Population of the study

The study included 100 adult volunteer patients diagnosed with nodular goiter. Conditions such as Graves’ disease or thyroiditis were not explicitly considered. Patients were selected randomly from walk-ins who fit the inclusion criteria. After meeting the criteria, participants were given a collection sheet to complete, which documented demographic and clinical details.

### 3.3. Design of study

This is a cross-sectional comparative study.

### 3.4. Area of study

This study was conducted in radiation and isotopes center Khartoum in Khartoum, Sudan.

### 3.5. Duration of the study

The duration of the study was from January 2021 to November 2021.

### 3.6. Sampling

The samples of this study were from 100 volunteer adult nodular goiter patients with differences in weight, height, age, and gender.

### 3.7. Machines

For nuclear medicine equipment & energy Window:

- Gamma camera: large field of view.
- Collimator: low energy, high resolution, parallel hole.
- Energy window: 20% window centered at 140 KeV.

Ultrasound was performed using a linear transducer with a frequency range of 6 to 15 MHz, selecting the highest frequency that still ensured sufficient sound wave penetration. The process began by positioning the transducer on the neck, adjusting the beam angle as needed, and fine-tuning the time gain compensation and sensitivity settings to achieve a consistent acoustic pattern for optimal thyroid imaging. Every patient was scanned with a standard ultrasound device (Toshiba Aplio 400, model SSA-370A), and the resulting images were recorded, printed, and stored.

## 4. Methods

### 4.1. Techniques

Thyroid scintigraphy was conducted 10 to 20 minutes following an intravenous dose of 37 to 111 MBq of sodium pertechnetate Tc-99m, utilizing a gamma-scintillation camera fitted with a low energy high-resolution collimator. Uptake measurements were based on the overall thyroid gland’s functional activity and classified as high, normal, or low. The study specifically refers to the uptake of the thyroid gland as a whole and not the uptake of individual nodules. A single Nuclear Medicine Physician evaluated all thyroid scintigraphy results. For standard ultrasound, every patient was positioned supine with their neck fully extended and scanned using a 7.5 to 10-MHz transducer. An expert radiologist performed neck ultrasounds on all participants. The collected data was processed and analyzed with the Statistical Package for Social Sciences software.

For the Doppler ultrasound scan, all patients were placed in a supine position with their necks stretched backward, employing a high-frequency linear-array transducer (6–15 MHz) to ensure sufficient depth and clear, detailed images. The examination covered both transverse and longitudinal views. Thyroid lesions were visualized in real time using a combination of gray-scale and color Doppler methods. Blood flow indices, including resistive index (RI), pulsatility index (PI), and peak systolic velocity (PSV), were measured using Doppler ultrasonography. PSV was obtained from the superior thyroid artery, and the insonation angle was maintained at ≤60° for optimal accuracy.

The features of a mass—such as its position, dimensions, form, edges, echogenicity, composition, and blood flow pattern—must be determined. No specific preparation is required if sedation isn’t used, but prior to administering the radiopharmaceutical:

The process should be clearly outlined to parents and to children who can comprehend it, with ongoing dialogue and comfort provided, explaining each phase to ensure cooperation and a smooth intravenous radiopharmaceutical injection.

A pre-sedation assessment is essential if sedation is planned (this includes obtaining informed consent, preparing the patient, and evaluating their condition for sedation administration).

Patients might need to stop thyroid medications 4 to 6 weeks prior to the scan, and adjustments may also be necessary for certain heart drugs or iodine-containing medications.

### 4.2. Data collection

**Figure 1.**
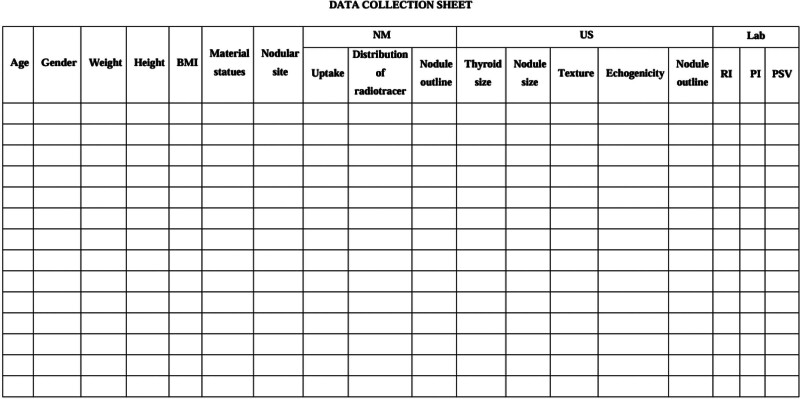
Data collection sheet provided to participants during the selection process, documenting inclusion criteria, demographics, and clinical details.

The data were collected using the following variables: thyroid morphology of both lobes, echogenetic texture and size (ultrasound measurements and gamma camera finding), age, gender, weight, and height, as documented in the data collection sheet provided to participants (Fig. 1). In patients with multiple nodules, the largest nodule was selected for measurement to represent the most prominent finding. Bilateral involvement was common, with nodules often present in both thyroid lobes. The selection process did not prioritize nodules based on suspicion of malignancy.

### 4.3. Image analysis

Thyroid uptake levels (high, normal, low) were assessed for the entire thyroid gland using Tc-99m scintigraphy.

A radiologist examined the results of both ultrasound and fine-needle aspiration biopsy.

### 4.4. Data analysis method

The Statistical Package for Social Sciences software was utilized to assess statistical significance through the application of a chi-square test.

### 4.5. Ethical considerations

The research was approved by the research licensing committee at the faculty of postgraduate studies at Nile University, Sudan. The permission was taken from the administrator of Asia Hospital before the beginning of data collection and verbal consent was taken from participants in case of agreement. All ethical considerations and participant privacy were kept. No participant identified was published.

## 5. Results

* We sampled 100 nodular goiter adult patients
* Demographic characteristics:
* *Age*: The study population consisted primarily of individuals aged 19 to 50, with a mean age of 39.26 years old, as recorded in the data collection sheet (see Fig. 1 for the data collection sheet used).
* The specific age distribution was as follows:

I. 19 to 35: 46%.
II. 36 to 50: 34%.
III. 51 to 65: 10%.
IV. Over 65: 10%.

**Figure 2.**
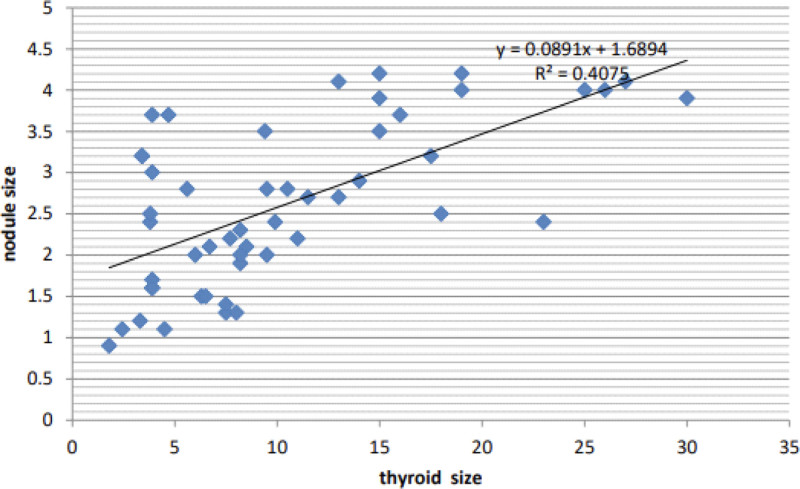
A scatter plot shows the relation between thyroid size and nodule size.

* *Gender*: A significant gender imbalance was observed, with 86% of participants being female and 14% being male.
* *Marital status*: The majority of participants were married (76%), while 24% were single. While marital status has no direct clinical relevance to thyroid imaging findings, its inclusion provides a comprehensive context for the study population.
* Thyroid nodule characteristics:
* *Nodular site*: Bilateral nodules were the most common, accounting for 62% of cases. Left lobe nodules were found in 28% of participants, and right lobe nodules in 10%.
* *Uptake*: A large proportion of individuals exhibited high uptake in nuclear medicine scans, with 74% having high uptake, 10% having normal uptake, and 16% having low uptake.
* *Distribution of radiotracer*: A majority of participants (62%) had heterogeneous distribution of radiotracer distribution, while 38% had homogeneous distribution.
* *Outline*: The majority of thyroid nodules were well-defined (92%), with 8% being ill-defined.
* *Texture*: Heterogeneous texture was more common than homogeneous texture, with 60% and 40% respectively.
* *Echogenicity*: Echogenicity was evaluated specifically in relation to thyroid nodules, not the entire thyroid gland. Most nodules were isoechoic (68%), followed by hypoechoic (20%) and hyperechoic (12%).
* Relationship between thyroid size and nodule size:
* A moderate positive correlation was observed between thyroid size and nodule size, with a correlation coefficient of 0.4075. This suggests that larger thyroids tend to have larger nodules, but other factors may also influence nodule size (Fig. 2).
* Gender differences:
* *Thyroid size*: Males had larger thyroids compared to females, with a mean thyroid size of 17.9 cm³ for males and 9.2 cm³ for females.
* *Nodule size*: Males also had larger nodules compared to females, with a mean nodule size of 3.4 cm³ for males and 2.4 cm³ for females.
* Impact of uptake on blood flow indices:

*Differences in blood flow indices*: Table 1 evaluates the relationships between thyroid gland uptake levels (classified as high, normal, and low) and blood flow indices (RI, PI, and PSV). Uptake here refers to the overall thyroid gland’s functional activity as measured by Tc-99m scintigraphy, not specific nodules. Significant differences were observed across uptake categories for RI, PI, and PSV (*P*-values: .000, .000, and .005, respectively).
*Higher blood flow indices with higher uptake*: Individuals with higher thyroid nodule uptake levels (high) generally exhibited higher values for RI, PI, and PSV compared to those with normal or low uptake levels.

**Table 1:** A statistical analysis of the relationship between blood flow indices (resistance index—RI, peristaltic index—PI, and peak systolic velocity—PSV) and thyroid nodule uptake levels (normal, high, low). Uptake reflects the overall functional activity of the thyroid gland as assessed by scintigraphy and is not specific to individual nodules.

Uptake		Normal	High	Low	Total	*P*-value
RI	Mean	0.41	0.59	0.3	0.53	.000
	Number	10	74	16	100	
	Std. deviation	0.2	0.14	0.09	0.18	
PI	Mean	0.74	0.85	0.49	0.78	.000
	Number	10	74	16	100	
	Std. deviation	0.14	0.2	0.06	0.22	
PSV	Mean	1	1.22	1.75	1.28	.005
	Number	10	74	16	100	
	Std. deviation	0.0	0.63	0.86	0.67	

PI = peristaltic index, PSV = peak systolic velocity, RI = resistance index.

*Mean values*:

*RI*: The mean RI for the high uptake group was significantly higher than the normal and low uptake groups (0.59 vs 0.41 and 0.3, respectively).
*PI*: Similar to RI, the mean PI for the high uptake group was significantly higher (0.85 vs 0.74 and 0.49).
*PSV*: The mean PSV for the high uptake group was also significantly higher (1.75 vs 1.00 and 1.22).

*Correlation between uptake and other variables*: A significant correlation was found between echogenicity and uptake, suggesting that hypoechoic nodules are more likely to have high uptake.

## 6. Discussion

This was an analytical comparative study aimed at studying the comparison between ultrasonography and scintigraphy findings in goiter patients, conducted in the radiation and isotopes center Khartoum in Khartoum state from January to November 2021, the samples of this study were 100 volunteer adult patients randomly selected.

The study results found the mean of age was (39.26 ± 14.19) (yrs.), the mean of weight was (65.14 ± 8.57) (kg), the mean of height was (1.64 ± 0.07) (m), the mean of body mass index was (24.26 ± 3.4) (kg/m^2^), the mean of thyroid size was (10.45 ± 7.03) (cm^3^), the mean of nodule size was (2.62 ± 0.98) (cm^3^), the mean of RI was (0.53 ± 0.18) and the mean of PI was (0.78 ± 0.22) [Table 2].

**Table 2:** Descriptive statistics.

	N	Minimum	Maximum	Mean	Std. deviation
Age (yrs.)	100	19	70	39.26	14.19
Weight (Kg)	100	48	85	65.14	8.57
Height (m)	100	1.50	1.9	1.64	0.07
BMI (Kg/m^2^)	100	18.0	33.5	24.26	3.4
Thyroid size (cm^3^)	100	1.80	30	10.45	7.03
Nodule size (cm^3^)	100	0.9	4.2	2.62	0.98
Resistance index	100	0.05	0.75	0.53	0.18
Peristaltic index	100	0.40	1.2	0.78	0.22

BMI= body mass index, N = number of patients.

The study results found that most patients in the age group (19–35) were (46%) then the age group (36–50) was (34%) and the other 2 groups were (10%) for each one. Regarding gender, the results showed most patients were females (86%) and males (14%). Most of the patients (76%) were married and (24%) were single. In most patients thyroid nodules affected both lobes was (62%), (28%) was affected in the left lobe and patients affected in right lobe was (10%).

The study results found from thyroid scintigraphy most patients had high uptake (74%), (16%) had low uptake, and (10%) had normal uptake. Regarding to distribution of radiotracer (62%) was homogeneous and (38%) was heterogeneous. In concern distribution of thyroid texture, 60% of cases exhibited a heterogeneous texture, while 40% exhibited a homogeneous texture. Heterogeneous texture refers to irregular or mixed echogenicity within the gland, which may indicate nodular changes, inflammation, or fibrosis. Homogeneous texture suggests uniform echogenicity, which is typically seen in a healthy gland or less advanced pathology.

Regarding echogenicity most patients showed isoechoic (68%), (20%) showed hypoechoic and (12%) showed hyperechoic. In concern distribution of nodule outlines (92%) look well-defined and (8%) look ill-defined.

Regarding to distribution of the PSV (peak systolic velocity) test the results showed peripheral was (84%), avascular was (12%) and central was (4%).

From the correlation between thyroid uptake in nuclear medicine and US findings, the results found there was an insignificant correlation between thyroid uptake and texture (*P*-value = .128) majority in heterogeneous high uptake was 48, and there was a significant correlation between thyroid uptake and echogenicity (*P*-value = .009) majority in isoechoic high uptake was 54 and there was insignificant correlation between thyroid uptake and nodular outline (*P*-value = .217) majority in well define high uptake was 66 [Table 3].

**Table 3:** Correlation between thyroid uptake in NM and US findings.

		Uptake			Total	*P*-value
		Normal	High	Low		
Texture	Heterogeneous	6	48	6	60	.128
	Homogeneous	4	26	10	40	
	Total	10	74	16	100	
Echogenicity	Hypoechoic	2	16	2	20	.009
	Hyperechoic	4	4	4	12	
	Isoechoic	4	54	10	68	
	Total	10	74	16	100	
Nodular outline	Well define	10	66	16	92	.217
	Ill define	0	8	0	8	
	Total	10	74	16	100	

NM = nuclear medicine, US = ultra-sound.

The study results found there was an insignificant difference in thyroid size regarding thyroid uptakes (*P*-value = .57) and an insignificant difference in nodule size rewarding to thyroid uptakes (*P*-value = .79). This indicates that the overall thyroid gland’s functional activity, as measured by total gland uptake in scintigraphy, does not significantly influence either the gland’s total volume or the size of its nodules. These findings align with the study’s observation of similar thyroid and nodule sizes across different uptake categories. The mean of nodule size was (2.44 ± 1.12) (cm^3^), (2.62 ± 0.94) (cm^3^) and (2.71 ± 1.11) (cm^3^) respectively for normal, high and low uptake [Table 4], agree with Mohammed^[3]^ who found the activity of the thyroid represented by thyroid uptake does not affect the volume. The research demonstrated a notable disparity in thyroid size between males and females, with a *P*-value of .009. The average thyroid size was 17.99 ± 9.02 cm³ for males and 9.22 ± 5.85 cm³ for females. Hsiao and colleagues found that males (n = 115) had greater thyroid volumes compared to females (n = 48), specifically 8.3 ± 3.3 mL versus 6.1 ± 2.6 mL (*P* < .001). and there was insignificant difference in nodule size between males and females (*P*-value = .801) the mean of thyroid size was (3.41 ± 0.87) (cm^3^) and (2.48 ± 0.94) (cm^3^), respectively. Agree with Hahn et al^[1]^ who found there were no significant differences in nodule size in age and gender.

**Table 4:** Comparison of thyroid gland size and the largest nodule size across uptake levels (high, normal, low). Uptake refers to the overall thyroid gland’s functional activity as measured by scintigraphy, and nodule size reflects the largest observed nodule in each patient.

Uptake		Thyroid size	Nodule size
Normal	Mean	10.96	2.44
	Number	10	10
	Std. deviation	8.24	1.12
High	Mean	10.75	2.62
	Number	74	74
	Std. deviation	7.19	0.94
Low	Mean	8.74	2.71
	Number	16	16
	Std. deviation	5.49	1.11
Total	Mean	10.45	2.62
	Number	100	100
	Std. deviation	7.03	0.98
*P*-value		.57	.79

The study results found there was a significant difference in blood flow indices regarding thyroid uptakes (*P*-value = .00, .00, and .005), respectively for RI, PI, and PSV. According to [Table 1] and Mohammed,^[3]^ the analysis of blood flow indices, including PI, RI, and PSV, using the ANOVA test on groups with varying thyroid uptakes, revealed that RI and PSV scores indicated significant effects. This suggests that blood flow is impacted by thyroid function in a manner consistent with normal behavior.

Finally, the study results found there was a positive relation between thyroid size and nodule size (thyroid size = 0.0891 * nodule size + 1.6894) (*R*² = 0.4075), as illustrated in the scatter plot (Fig. 2), agreeing with Mohammed^[3]^ who found there is a correlation between thyroid nodule volume and thyroid volume.

While our study provides valuable insights into the role of ultrasonography and thyroid scans in goiter management, it is important to acknowledge certain limitations. The observational design of the study limits our ability to establish direct cause-and-effect relationships.

The inclusion of marital status in this study was intended to provide a comprehensive demographic context. While not directly relevant to the comparison of imaging findings, marital status could indirectly correlate with lifestyle or health factors influencing thyroid conditions, such as stress levels—associated with thyroid dysfunction—and dietary habits, including iodine intake, which may differ between single and married individuals.

Furthermore, inter-observer variability in image interpretation and the inherent subjectivity involved in assessing thyroid characteristics may also affect the accuracy of the results. The study was conducted in Khartoum, Sudan, and the generalizability of the findings to other geographic regions may be limited.

Our findings suggest that both ultrasonography and thyroid scan provide valuable information for the diagnosis and management of goiter. Ultrasonography is particularly useful for assessing thyroid size, nodule characteristics, and blood flow indices, This approach can aid in pinpointing patients with an elevated risk of complications and inform treatment choices.

A thyroid scintigraphy scan is essential for evaluating thyroid function and identifying areas of abnormal radiotracer uptake, providing valuable information for diagnosis and monitoring.

The combined use of ultrasonography and thyroid scintigraphy scans can offer a more comprehensive assessment of goiter patients, aiding in diagnosis, staging, and treatment planning. Regular follow-up with both modalities can help monitor disease progression, evaluate treatment response, and detect early signs of complications.

When deciding between ultrasonography and thyroid scan, clinicians should carefully consider the potential benefits and costs of the individual patient’s clinical presentation and risk factors.

Further research is needed to explore the long-term implications of these findings and to investigate the cost-effectiveness of using ultrasonography and thyroid scan in combination or individually. Additionally, studies investigating the role of emerging imaging techniques, such as ultrasound elastography or molecular imaging, in the evaluation of goiter would be valuable.

## 7. Conclusion

Both ultrasonography and thyroid scintigraphy provide essential but distinct insights for managing nodular goiter. In resource-limited settings, ultrasonography, enhanced by Doppler blood flow analysis, can serve as a primary diagnostic tool. Scintigraphy should be reserved for cases requiring functional assessment, such as suspicious uptake patterns or inconclusive ultrasonography. Future studies should explore integrating advanced ultrasonographic techniques, such as elastography, to further refine diagnostic accuracy.

## 8. Recommendations

The study recommends:

* *Comprehensive thyroid evaluation*: Employ a combination of ultrasound and thyroid scintigraphy scans for a thorough evaluation of thyroid disorders, enabling early detection, monitoring of treatment response, and identification of recurrence.
* *Thorough patient history and assessment*: Prior to initiating any thyroid scan, gather a detailed patient history to identify relevant risk factors and symptoms, aiding in the interpretation of imaging findings and guiding subsequent management.
* *Operator expertise and equipment*: Ensure that ultrasound examinations are performed by skilled operators with specialized training to guarantee accurate and reliable results. Invest in high-resolution ultrasound equipment with advanced features, such as Doppler capabilities, to enhance diagnostic accuracy.
* *Infrastructure development*: Governments and healthcare organizations should prioritize the expansion of nuclear medicine centers to address the growing need for thyroid imaging services, especially in regions with limited access. Allocate adequate funding and resources to ensure the availability of these essential facilities.
* *Public health initiatives*: Promote public awareness about thyroid health through educational campaigns, encouraging regular checkups and early detection. Implement iodine supplementation programs and encourage a balanced diet rich in iodine-containing foods to reduce the prevalence of thyroid diseases.
* *Continued research*: Ongoing research is essential to advance our understanding of thyroid nodule biology, develop innovative diagnostic and therapeutic approaches, and improve patient outcomes. Collaborate with researchers, clinicians, and policymakers to drive progress in this field.

## Acknowledgments

My thanks to Dr Alaa Eldeen Mohammed Abdalgaioum who devoted her time and generously gave her knowledge and experience to me without limits and agreed to be named in this research. My thanks extended to RICK Hospital which is the place where we took all samples.

## Author contributions

**Conceptualization:** Fares Abboud, Hind Alsiddig.

**Data curation:** Hind Alsiddig.

**Formal analysis:** Fares Abboud, Hind Alsiddig.

**Investigation:** Fares Abboud, Hind Alsiddig.

**Methodology:** Hind Alsiddig.

**Project administration:** Fares Abboud, Hind Alsiddig.

**Supervision:** Hind Alsiddig.

**Validation:** Fares Abboud.

**Visualization:** Fares Abboud.

**Writing – original draft:** Fares Abboud, Hind Alsiddig, Sultaneh Haddad, Basil Daradkeh, Ghassan Bayat, Farah Haneyah.

**Writing – review & editing:** Fares Abboud, Hind Alsiddig, Sultaneh Haddad, Basil Daradkeh, Ghassan Bayat, Farah Haneyah.
